# Edaravone Mitigates Postovulatory Aging by Preserving Oocyte and Embryo Quality in Mice

**DOI:** 10.3390/antiox14101215

**Published:** 2025-10-09

**Authors:** Kyeoung-Hwa Kim, Eun-Young Kim, Ah-Reum Lee, Mi-Kyoung Koong, Kyung-Ah Lee

**Affiliations:** 1 CHA University Global IVF Group, Pangyo-ro 335, Bundang-gu, Seongnam-si 13488, Republic of Korea; khkim@chamc.co.kr (K.-H.K.); keyovary@chamc.co.kr (E.-Y.K.); rearum@chamc.co.kr (A.-R.L.); 2 CHA University Fertility Center Daegu, Dalgubeol-daero 2095, Jung-gu, Daegu 41936, Republic of Korea; mkkoong1@chamc.co.kr; 3 Department of Biomedical Science, College of Life Science, CHA University, Pangyo-ro 335, Bundang-gu, Seongnam-si 13488, Republic of Korea

**Keywords:** Edaravone, postovulatory aging, oocyte quality, antioxidant effect, reproductive safety

## Abstract

Postovulatory aging (POA) significantly contributes to fertility decline, primarily through oxidative stress, which impairs oocyte quality, reduces embryonic developmental competence, and may adversely affect offspring health. Edaravone (EDA), a potent free radical scavenger, is known for its cytoprotective effects in various disease models. This study aimed to evaluate whether EDA can mitigate the detrimental effects of POA on mouse oocyte and embryo quality and confirm its reproductive safety. Supplementation with 10 nM EDA significantly reduced meiotic abnormalities, restored mitochondrial distribution, enhanced mitochondrial membrane potential and ATP production, and decreased intracellular reactive oxygen species (ROS) in aged oocytes. Although EDA did not markedly improve fertilization or blastocyst formation rates, it enhanced embryo quality, with morphokinetic parameters comparable to those of young oocytes. Moreover, F_1_ offspring derived from embryos produced by EDA-treated POA oocytes were healthy, and female progeny exhibited normal reproductive competence. These findings demonstrate that EDA safely improves oocyte quality by alleviating POA-induced oxidative damage, offering a potential antioxidant strategy to enhance assisted reproductive technology (ART) outcomes when applied to IVF clinics.

## 1. Introduction

Compromised oocyte quality significantly contributes to reduced reproductive potential, leading to subfertility and infertility. This deterioration results from multiple factors, including chronological aging, lifestyle, environmental toxins, genetic predisposition, and physiological stressors [1]. Postovulatory aging (POA) occurs when oocytes, arrested at the metaphase II (MII) stage after ovulation, remain unfertilized, either in vivo (within the oviduct) or in vitro (in culture medium). This time-dependent process progressively impairs oocyte quality [2]. Low-quality oocytes exhibit spindle and chromosomal aberrations, mitochondrial dysfunction, elevated oxidative stress, epigenetic alterations, disrupted calcium homeostasis, defective cortical granule exocytosis, zona hardening, and impaired sperm binding. Consequently, POA reduces fertilization efficiency, compromises embryo development, causes implantation failure, and may lead to developmental abnormalities in offspring [3]. To address these issues, studies have employed in vitro models of postovulatory-aged oocytes supplemented with antioxidants or other compounds to restore oocyte quality and delay aging [4,5,6,7].

While the physiological consequences of POA are well-documented, its molecular mechanisms are not fully elucidated. Oxidative stress plays a central role in oocyte quality deterioration during POA [3,8]. Increased reactive oxygen species (ROS) production accompanies both POA and chronological aging. In mice, antioxidants such as auraptene, coenzyme Q10, melatonin, resveratrol, and N-acetyl-cysteine have been shown to mitigate ROS levels and improve oocyte quality during POA [4,6,7,9,10]. In humans, POA is particularly critical in assisted reproductive technology (ART) procedures, where oocytes are cultured for extended periods before in vitro fertilization (IVF) due to laboratory constraints or delayed semen collection, compromising embryo viability. Thus, strategies to delay or prevent POA are essential for improving ART outcomes.

Edaravone (EDA, 3-methyl-1-phenyl-2-pyrazolin-5-one), a potent free radical scavenger, is clinically approved for oxidative stress-related conditions, including ischemic stroke and amyotrophic lateral sclerosis (ALS). Marketed as Radicava in countries such as Japan, South Korea, and the United States, EDA exhibits antioxidative, anti-inflammatory, anti-apoptotic, anti-fibrotic, and neuroprotective properties, and restores mitochondrial function [11,12,13,14,15,16]. However, its effects on oocyte quality during POA and chronological aging, as well as on embryo development, remain largely unexplored. Furthermore, its safety and efficacy in reproductive medicine require further investigation.

This study aimed to determine whether EDA supplementation could enhance oocyte quality in postovulatory-aged and chronologically aged oocytes and improve embryonic developmental competence. Additionally, we assessed the efficacy and safety of EDA on reproductive and developmental outcomes in first-generation (F_1_) offspring, including viability, morphological normality, and female fertility.

## 2. Materials and Methods

### 2.1. Animals

ICR and BDF1 mice were obtained from Koatech (Pyeongtaek, Republic of Korea). Female 4-week-old ICR mice were primarily used in this study, while BDF1 mice were used for IVF, time-lapse imaging, and live birth safety assays. Female 6-month-old C57BL/6 mice were sourced from Jabio (Suwon, Republic of Korea) and maintained for an additional 6 months at the Laboratory Animal Research Center of CHA University to reach 12 months of age for experiments investigating EDA’s effects on aging-associated meiotic defects. All procedures were conducted in accordance with institutional guidelines and approved by the Institutional Animal Care and Use Committee of CHA University. Different mouse strains were used according to experimental needs: ICR mice for oocyte experiments, BDF1 mice for embryo transfer and coat color-based identification of offspring, and thus also used in fertilization and embryo development experiments, and aged C57BL/6 mice for evaluating age-related meiotic defects due to the limited availability of aged ICR mice. All procedures were conducted in accordance with institutional guidelines and approved by the Institutional Animal Care and Use Committee of CHA University.

### 2.2. Reagents and Antibodies

Unless otherwise specified, chemicals, reagents, and media were purchased from Sigma-Aldrich (St. Louis, MO, USA). Edaravone (M70800) was dissolved in dimethyl sulfoxide (DMSO) and diluted to 10 nM in M16 medium (M7292). Mouse anti-α-tubulin antibody (sc-8035) was obtained from Santa Cruz Biotechnology (Dallas, TX, USA).

### 2.3. MII Oocyte Collection and Postovulatory Aging

The MII oocyte isolation and ovarian stimulation were performed as previously described [4]. Briefly, 4-week-old female ICR mice were injected intraperitoneally with 7.5 IU pregnant mare’s serum gonadotropin (PMSG; Daesung, Uiwang, Republic of Korea), followed by 7.5 IU human chorionic gonadotropin (hCG; CG5) 48 h later. After 14–16 h, cumulus–oocyte complexes (COCs) were collected, and cumulus-free MII oocytes were obtained using hyaluronidase. For the young group, MII oocytes were used immediately after cumulus cell removal. For the POA group, MII oocytes were cultured in M16 medium containing 1% penicillin–streptomycin (Gibco, Grand Island, NY, USA) for 12 h at 37 °C in 5% CO_2_ to induce postovulatory aging. For the POA + EDA group, MII oocytes were cultured in M16 medium supplemented with 10 nM EDA for 12 h under the same conditions.

### 2.4. Germinal Vesicle (GV) Oocyte Collection and In Vitro Maturation (IVM)

GV oocytes were isolated from 12-month-old female C57BL/6 mice 46 h after PMSG injection. To prevent meiotic progression during collection, M2 medium (M7167) containing 0.2 mM 3-isobutyl-1-methylxanthine (IBMX) was used. Ovaries were dissociated in M2 medium with IBMX, and cumulus cells were mechanically removed from COCs to obtain denuded GV oocytes. For IVM, GV oocytes were cultured in M16 medium with or without 10 nM EDA (Aged and Aged + EDA groups, respectively) for 14–16 h at 37 °C in 5% CO_2_. Young oocytes from 4-week-old female C57BL/6 mice were cultured under the same conditions for comparison. Post-IVM, oocyte maturation was assessed by the presence of a germinal vesicle (GV oocyte), a polar body (MII oocyte), or neither (MI oocyte).

### 2.5. Immunofluorescence Staining

Mitochondrial distribution was assessed using MitoTracker Orange CMTMRos (M7510; Molecular Probes, Eugene, OR, USA). Oocytes were incubated in M16 medium with 300 nM MitoTracker for 30 min at 37 °C in 5% CO_2_. After washing with phosphate-buffered saline containing 0.1% polyvinyl alcohol (PBS-PVA), oocytes were fixed and permeabilized in PBS-PVA with 3.7% paraformaldehyde and Triton X-100 for 40 min at room temperature. Following blocking, oocytes were stained overnight at 4 °C with anti-α-tubulin antibody, followed by incubation with Alexa Fluor 488-conjugated secondary antibody (A11001; Thermo Fisher Scientific, Waltham, MA, USA). DNA was stained with DAPI, and oocytes were mounted on glass slides. Mitochondrial distribution, spindle organization, and chromosome alignment were observed using a Leica confocal microscope (TCS SP5 II; Wetzlar, Germany). At least 45 oocytes per group were analyzed across three replicates.

### 2.6. Measurement of Mitochondrial Membrane Potential (ΔΨm) and ATP Levels

For evaluation, the oocytes were stained with MitoProbe JC-1 (68-0851-38, Invitrogen, Carlsbad, CA, USA). Oocytes were cultured in M16 medium supplemented with JC-1 at 1 µg/mL for 30 min and then washed with PBS-PVA. The oocytes were mounted and immediately imaged in the red and green fluorescence channels under a Leica confocal microscope. For quantitative analysis, Leica Application Suite Advanced Fluorescence Lite software (LAS AF Lite 2.3.5 version) was used to measure the signal intensities, and ΔΨm was calculated as the ratio of the red/green signals.

The levels of ATP in the oocytes were measured using a BODIPY FL ATP (A12410, Molecular Probes, Eugene, OR, USA). MII oocytes were cultured in M16 medium containing 500 nM BODIPY FL ATP for 1 h in the dark. After washing with PBS-PVA, the oocytes were fixed, permeabilized, and mounted on glass slides. Fluorescence signals were measured using a Leica confocal microscope. For the intracellular ATP measurements, the signal intensities for each oocyte from different groups were calculated using LAS AF software.

### 2.7. Measurement of Intracellular ROS and Glutathione (GSH) Levels

Intracellular ROS and GSH levels were measured using CM-H2DCFDA (C6827; Invitrogen) and ThiolTracker Violet (T10095; Invitrogen), respectively. Oocytes were incubated in M16 medium with 5 mM CM-H2DCFDA or 20 mM ThiolTracker Violet for 30 min at 37 °C in 5% CO_2_ in the dark. After mounting, fluorescence signals were quantified using a Leica confocal microscope and LAS AF software.

### 2.8. IVF and Preimplantation Embryo Culture

Sperm were collected from the cauda epididymis of 8- to 12-week-old BDF1 male mice, placed in EmbryoMax human tubal fluid medium (MR-070) covered with mineral oil, and incubated for 1 h at 37 °C in 5% CO_2_ for capacitation. After 12 h of aging for the POA and POA + EDA groups, MII oocytes from all groups (young, POA, POA + EDA) were fertilized in HTF droplets with 4 × 10^5^ sperm/mL for 6 h at 37 °C in 5% CO_2_. Oocytes were washed to remove sperm and cultured in EmbryoMax KSOM mouse embryo medium (MR-121) for up to 5 days. Embryo developmental stages were examined under a microscope.

### 2.9. Time-Lapse Imaging System

Embryo development was monitored using a time-lapse microscope (iEM900; CNC Biotech, Seoul, Republic of Korea). Zygotes with two pronuclei were cultured, and images were captured every 5 min for 120 h. Sequential images were converted into movie files, and morphokinetic parameters were recorded: t0 (insemination), tf (pronuclei fading and entry into first embryonic M-phase), t2–t8 (hours post-insemination for 2- to 8-cell stages), tErB (start of blastocoel cavity formation), tBL (half or more of blastocoel cavity formed), CC (cell cycle length), and S (synchronicity of cleavage division.

### 2.10. Live Birth Safety Assay

Blastocysts were cultured in EmbryoMax KSOM mouse embryo medium for 5 days and transferred at the early blastocyst stage to 2.5 days post coitum (dpc) pseudo-pregnant ICR female mice (8–10 weeks old) mated with vasectomized males. Five blastocysts from the young, POA, and POA + EDA groups were transferred to each uterine horn. Offspring were delivered at 19.5 days of gestation, nursed by lactating ICR female mice, and monitored until 8 weeks of age.

Fertility of female offspring was assessed by injecting 8-week-old offspring with 7.5 IU PMSG, followed by 7.5 IU hCG 48 h later. Ovulated oocytes were collected after 14–16 h and analyzed for oocyte number, morphology, cytoplasmic mitochondrial distribution, and chromosomal/spindle abnormalities.

### 2.11. Data Analysis and Statistics

Experiments were repeated at least three times unless otherwise indicated. Data are presented as mean ± standard error of the mean (mean ± SEM). Statistical comparisons among multiple groups were performed using one-way ANOVA. Differences with *p* < 0.05 were considered significant.

## 3. Results

### 3.1. EDA Alleviates Meiotic Defects in Postovulatory-Aged Oocytes

**Figure 1:**
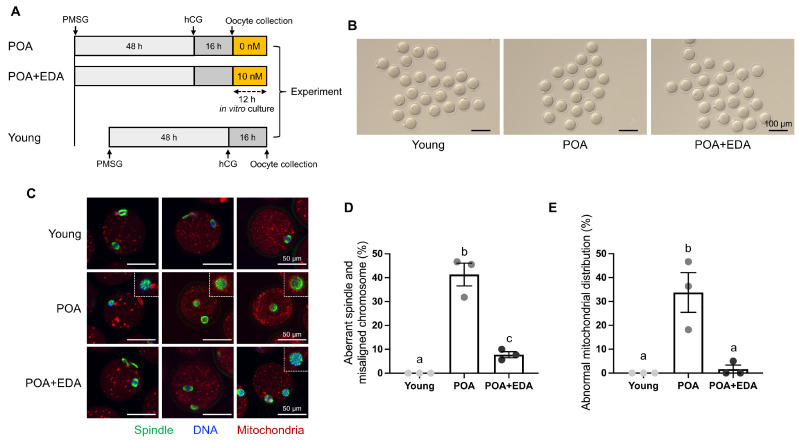
EDA ameliorates meiotic defects in oocytes during postovulatory aging: (**A**) timeline scheme for hormone priming, postovulatory aging, and EDA supplementation to investigate how EDA impacts postovulatory-aged oocytes. (**B**) Micrographs of MII oocytes in young (young) and postovulatory-aged oocytes treated without (POA) or with the EDA (POA + EDA). The scale bars represent 100 μm. (**C**) Representative image of meiotic spindle morphology and chromosome alignment in MII oocytes from each group. Images illustrate a single individual oocyte. Green, spindle; blue, chromosome; red, mitochondria; white dashed-line small box, disrupted spindle and misaligned chromosomes. The scale bars represent 50 μm. (**D**) The proportion of MII oocytes with meiotic defects in each group. The data are expressed as the means ± SEM. Dots of bars in each group show individual values of three independent experiments. Different letters indicate significant differences at *p* < 0.05. (**E**) The percentage of abnormal mitochondrial distribution was quantified in each group. Different letters indicate significant differences at *p* < 0.05.

Meiotic spindle morphology and chromosome distribution are critical indicators of oocyte quality. To investigate whether EDA ameliorates the detrimental effects of POA, MII oocytes were cultured in vitro with or without 10 nM EDA during POA (Figure 1A). The concentration of 10 nM EDA was selected based on preliminary experiments, in which 1 nM EDA did not improve the reduced fertilization rate and embryonic development induced by POA, and 100 nM EDA showed reduced efficacy compared to 10 nM EDA (Figure S1). Among the tested concentrations, 10 nM EDA showed the most beneficial effects on oocyte quality and embryonic development. Spindle assembly, chromosomal organization, and mitochondrial distribution were then assessed. Postovulatory-aged MII oocytes, with or without EDA (POA and POA + EDA groups, respectively), appeared morphologically normal compared to young oocytes (Figure 1B). Young MII oocytes exhibited barrel-shaped spindles with neatly aligned chromosomes (Figure 1C,D). In contrast, POA oocytes displayed irregular microtubule architecture and misaligned chromosomes despite normal MII morphology (Figure 1C,D). Notably, the POA + EDA group showed significantly reduced meiotic defects, with improved spindle organization and chromosome alignment compared to the POA group (41.3% vs. 7.7%; Figure 1C,D).

In the young group, MII oocytes had evenly distributed mitochondria in the cytoplasm (Figure 1C,E). Conversely, POA oocytes exhibited abnormal mitochondrial accumulation around the meiotic spindle or throughout the cytoplasm (Figure 1C,E). These abnormalities were markedly reduced in the POA + EDA group (33.7% vs. 1.7%; Figure 1E). These findings suggest that EDA promotes proper spindle and chromosome architecture and restores mitochondrial distribution in postovulatory-aged MII oocytes.

### 3.2. EDA Ameliorates Aging-Associated Meiotic Defects in Oocytes

**Figure 2:**
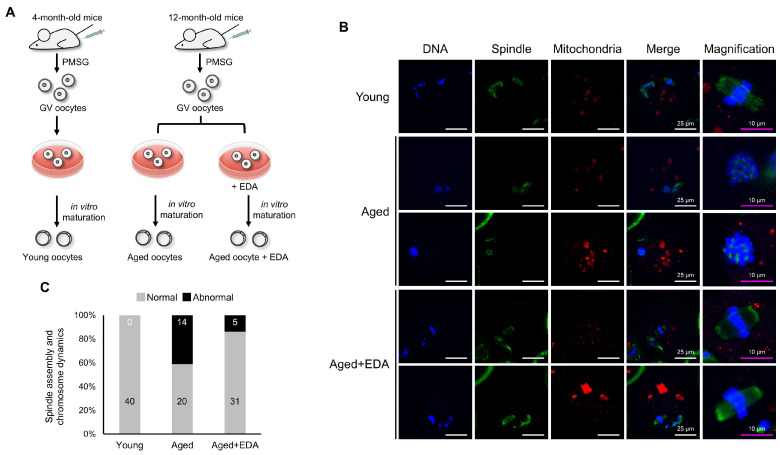
EDA also prevents meiotic defects in naturally aged oocytes: (**A**) schematic representation showing the experimental design to investigate how EDA impacts naturally aged oocytes. (**B**) Representative image of meiotic spindle morphology and chromosome alignment in MII oocytes in young and naturally aged oocytes treated without (Aged) or with EDA (Aged + EDA). Young, oocytes obtained from 4-week-old female mice; Aged, oocytes obtained from 12-month-old female mice. Green, spindle; blue, chromosome; red, mitochondria. The white scale bars (in the DNA, spindle, mitochondria, and merge columns) represent 25 μm, and pink scale bars (in the magnification column) represent 10 μm. (**C**) Bar graphs represent the percentage of MII oocytes with barrel-like spindles and well-aligned chromosomes (gray) and abnormal-shaped spindles and misaligned chromosomes (black), quantified for young and maternally aged oocytes treated without or with EDA. The numbers in the bar show the number of MII oocytes.

Maternal aging impairs spindle assembly and chromosomal organization in oocytes. We next examined whether EDA can restore the deleterious meiotic defects caused by maternal aging. Aged GV oocytes from 12-month-old female mice were cultured in medium treated with or without 10 nM EDA, and meiotic progression was analyzed (Figure 2A). To examine whether EDA mitigates aging-associated meiotic defects, GV oocytes from 12-month-old female mice were cultured with or without 10 nM EDA, and meiotic progression was analyzed (Figure 2A). Aged oocytes exhibited disrupted spindles and misaligned chromosomes (41.2%; Figure 2B,C). Notably, EDA supplementation during in vitro maturation significantly reduced these defects (13.9%; Figure 2B,C), with oocytes in the Aged + EDA group displaying normal bipolar spindles and well-aligned chromosomes at the equatorial plane (Figure 2C). These results indicate that EDA suppresses age-related meiotic defects, highlighting its antioxidant potential in mitigating maternal aging effects.

### 3.3. EDA Reverses Mitochondrial Dysfunction and Mitigates Oxidative Stress in Postovulatory-Aged Oocytes

**Figure 3:**
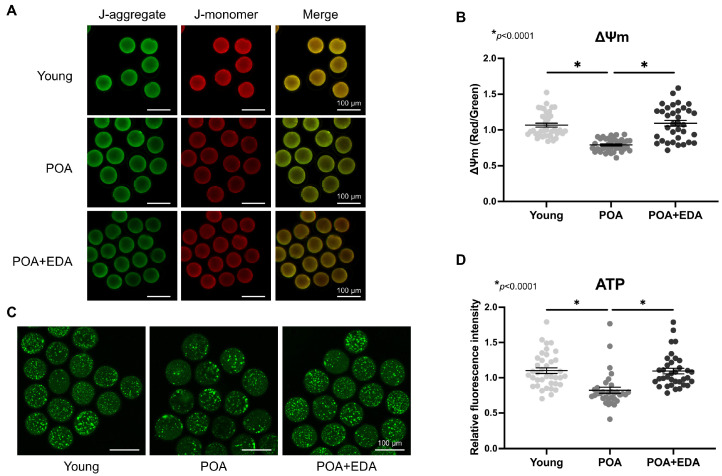
EDA improved mitochondrial function in postovulatory aging oocytes: (**A**) Representative images of ΔΨm in postovulatory-aged MII oocytes supplemented with (POA + EDA group) or without EDA (POA group). ΔΨm indicates the ratio of RITC (J-aggregate, high membrane potential) to FITC (J-monomer, low membrane potential) intensity in oocytes. Scale bars represent 100 μm. (**B**) Scatter dot plot with means ± SEM showing quantified ΔΨm from the data in (**A**). Each dot represents an individual value of ΔΨm. The asterisks represent statistical significance at *p *< 0.0001. (**C**) Representative images of ATP levels in young and postovulatory-aged oocytes without or with EDA supplementation. Scale bars represent 100 μm. (**D**) Scatter dot plot with means ± SEM showing quantitated ATP from the data in (**C**). Each dot represents an individual value of ATP signals. The asterisks represent statistical significance at *p *< 0.0001.

Mitochondria are pivotal indicators of oocyte quality, essential for maturation, fertilization, and embryonic development. The effects of EDA on mitochondrial function were assessed using JC-1 and BODIPY FL ATP staining. Red fluorescence indicates high mitochondrial membrane potential (ΔΨm), while green fluorescence indicates low ΔΨm. The red/green intensity ratio was reduced in POA oocytes but significantly increased in the POA + EDA group, comparable to the young group (Figure 3A,B). ATP levels were also reduced in POA oocytes compared to young oocytes, but they were restored by EDA treatment (Figure 3C,D).

**Figure 4:**
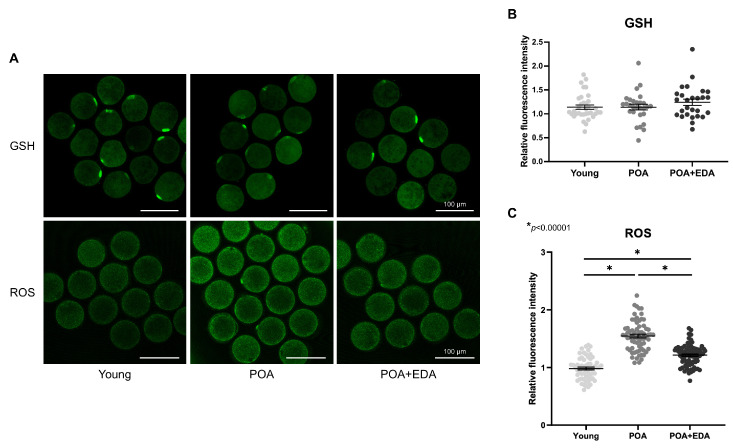
EDA reduced intracellular ROS in postovulatory-aged oocytes: (**A**) Representative image of intracellular levels and distribution of GSH and ROS in the young, POA, and POA + EDA groups. Scale bars represent 100 μm. (**B**) The fluorescence intensity of GSH signals was quantified in each group. The data are presented as the means ± SEM. Each dot represents an individual value of GSH signals. (**C**) The fluorescence intensity of signals from intracellular ROS was quantified in each group. The data are presented as the means ± SEM. Each dot represents an individual value of ROS signals. The asterisks represent statistical significance at *p *< 0.00001.

Mitochondrial dysfunction is associated with elevated oxidative stress, compromising oocyte quality. Intracellular GSH levels remained unchanged in POA oocytes (Figure 4A, upper panel; Figure 4B), whereas ROS levels were significantly elevated in POA oocytes and reduced in the POA + EDA group (Figure 4A, lower panel; Figure 4C). These findings suggest that EDA restores mitochondrial function and reduces oxidative stress in postovulatory-aged oocytes by modulating mitochondrial activity.

### 3.4. EDA Partially Restores Embryo Developmental Competence in Postovulatory-Aged Oocytes

**Figure 5:**
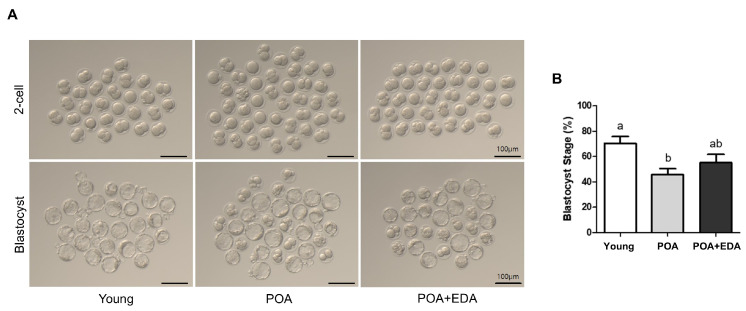
Modest improvement by EDA in fertilization and embryonic developmental competence of postovulatory-aged oocytes: (**A**) Representative images of 2-cell stage embryos and blastocyst embryos derived from the young, aged, and EDA-treated aged oocytes. Scale bars represent 100 μm. (**B**) Quantitative analysis of blastocyst formation rate. The data are presented as the means ± SEM, and at least five independent experiments were performed. Different letters indicate significant differences at *p* < 0.05.

Oocyte quality is critical for fertilization and embryonic development. To evaluate EDA’s effects on fertilization and developmental potential, IVF was performed. The 2-cell embryo formation rate, indicative of fertilization, was lower in the POA group (62.0%) compared to the young group (76.8%; Table 1, Figure 5A). The POA + EDA group showed a slight improvement (69.0%), though not statistically significant compared to the young group (Table 1). Similarly, blastocyst formation was reduced in the POA group (71.2%) compared to the young group (89.2%; Table 1, Figure 5A,B) but improved in the POA + EDA group (78.2%), indicating partial restoration of developmental competence (Table 1).

**Figure 6:**
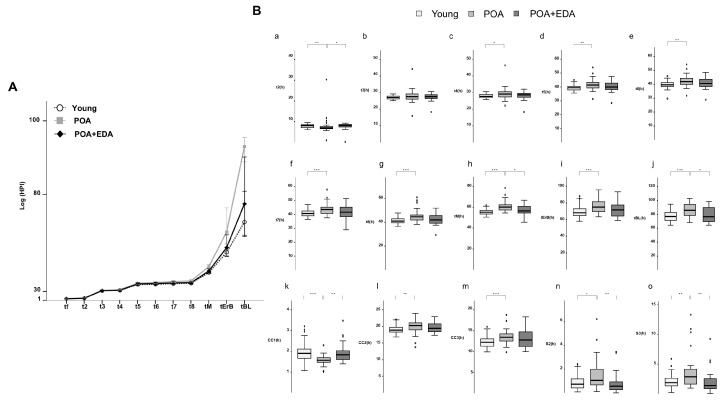
EDA improved the development of embryos derived from postovulatory-aged oocytes after IVF: (**A**) Comparison of the morphokinetics of embryos in the young, POA, and POA + EDA groups. (**B**) Time-lapse analysis of individually cultured embryos in each group. The bar plots represent the average time of each embryo’s developmental events. (**a**–**j**) t2, t3, t4, t5, t6, t7, t8, tM, tErB, and tBL represent the times to reach the 2-cell, 3-cell, 4-cell, 5-cell, 6-cell, 7-cell, 8-cell, morula, cavitating blastocyst, and blastocyst stages, respectively. (**k**–**m**) CC1, CC2, and CC3 represent the length of the first, second, and third cell cycles, respectively. (**n**,**o**) S2 and S3 represent the synchronicity or timing of the second and third rounds of cleavage division, respectively. The data are presented as the means ± SEM of five biologically independent experiments. * *p* < 0.05, ** *p* < 0.01, *** *p* < 0.001.

Using a time-lapse imaging system, embryo morphokinetics were analyzed. Embryos in the POA group developed to the blastocyst stage more slowly than those in the young and POA + EDA groups (Figure 6A). Morphokinetic parameters in the POA + EDA group were comparable to those of the young group (Figure 6B(a–o)). POA embryos exhibited delayed cleavage, cavitation, and blastocyst formation (Figure 6B(c–j,l–o)), whereas EDA treatment shortened tM and tBL parameters (Figure 6B(h,j)). Interestingly, the time to reach the 2-cell stage was shorter in the POA group than in the young and POA + EDA groups (Figure 6B(a,k)). These results suggest that EDA-treated POA oocytes produce embryos with morphokinetic parameters similar to those of young oocytes, indicating improved blastocyst quality.

### 3.5. Safety and Efficacy of EDA on Female Fertility of F_1_ Offspring

**Figure 7:**
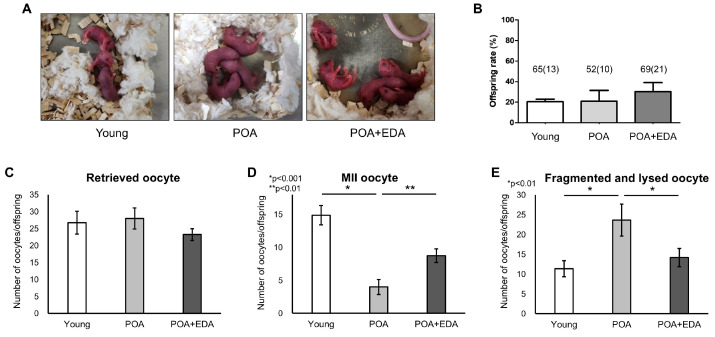
No harmful effects of EDA on the live birth rate of F_1_ mice and reproductive health of female F_1_ offspring: (**A**) Representative image of 2-day-old pups delivered by female mice in each group following embryo transfer. (**B**) Quantitative analysis of live birth rate of F_1_ mice. The total number of transferred blastocyst embryos and the number of offspring in parentheses are indicated. The data are presented as the means ± SEM. (**C**) The number of retrieved oocytes from female F_1_ offspring after superovulation was counted in the young, POA, and POA + EDA groups. (**D**) Numbers of MII oocytes were counted in each group from female F_1_ offspring. The results are presented as the means ± SEM. * *p* < 0.001, ** *p* < 0.01. (**E**) Numbers of fragmented and lysed oocytes were counted in each group from female F_1_ offspring. The results are presented as the means ± SEM. The asterisks represent statistical significance at *p *< 0.01.

To assess EDA’s safety and efficacy, blastocysts from the young, POA, and POA + EDA groups were transferred into recipient mice. Live birth rates and morphology of F1 offspring showed no significant differences across groups (20.0% vs. 19.2% vs. 30.4%; Figure 7A,B). Oocyte quality and fertility of female F1 offspring were evaluated after superovulation. The number of retrieved oocytes per female was similar across groups (26.8 oocytes vs. 28.0 oocytes vs. 23.3 oocytes; Figure 7C). However, MII oocyte numbers were significantly reduced in the POA group (4.0 oocytes) compared to the young (14.9 oocytes) and POA + EDA (8.8 oocytes) groups (Figure 7D). Fragmented and lysed oocytes were significantly more prevalent in the POA group (23.7 oocytes) than in the young (11.4 oocytes) and POA + EDA (14.2 oocytes) groups (Figure 7E).

**Figure 8:**
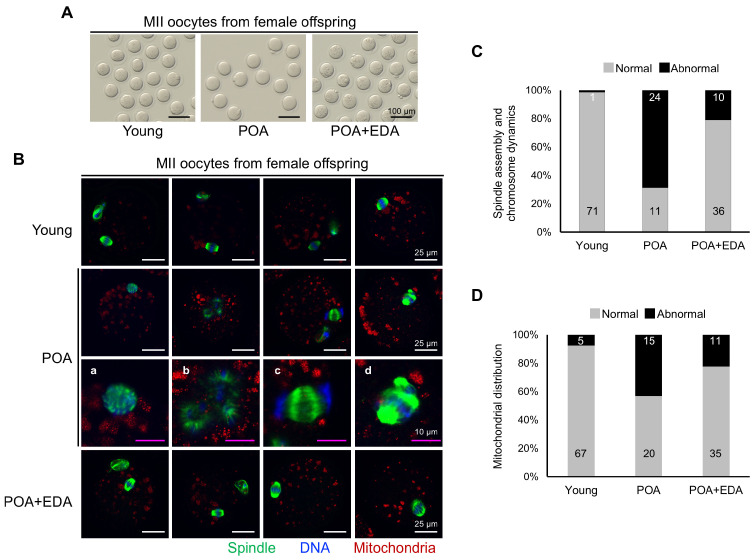
EDA ameliorates meiotic defects in oocytes of female F_1_ offspring derived from postovulatory-aged oocytes: (**A**) Micrographs of retrieved MII oocytes from female F_1_ offspring in the young, POA, and POA + EDA groups. The scale bars represent 100 μm. (**B**) Representative image of meiotic spindle morphology and chromosome arrangement in MII oocytes from female F_1_ offspring in each group. (a–d) Enlarged images of chromosomes and spindle apparatus in POA group. The white scale bars represent 25 μm, and pink scale bars in the magnification column of the POA group represent 10 μm. (**C**) Graph representing the percentage of MII oocytes with barrel-shaped spindle and well-aligned chromosome (gray) and abnormally shaped spindle and misaligned chromosome (black), quantified from female F_1_ offspring in the young, POA, and POA + EDA groups. The numbers in the bar show the number of MII oocytes. (**D**) The graph representing the percentage of MII oocytes with normal (gray) and abnormal mitochondrial distribution (black) was quantified in each group. The numbers in the bars show the number of MII oocytes.

Morphological analysis of MII oocytes showed no significant differences (Figure 8A). However, oocytes from POA group female offspring exhibited aberrant spindle structures and misaligned chromosomes (Figure 8B), including clustered spindles (Figure 8B(a)), dispersed spindles and chromosomes (Figure 8B(b)), mislocalized chromosomes (Figure 8B(c)), or multiple spindle–chromosome structures (Figure 8B(d)). These abnormalities were reduced in the POA + EDA group (Figure 8B,C). Abnormal mitochondrial distribution was also prevalent in the POA group but restored in the POA + EDA group (Figure 8D). These findings indicate that EDA is safe and supports reproductive fitness in F_1_ offspring.

## 4. Discussion

Since the birth of the world’s first IVF baby in 1978, IVF has become a cornerstone of ART for treating infertility and subfertility [17]. However, delayed fertilization during ART can trigger POA, compromising oocyte quality, fertilization potential, and embryo developmental competence, thus reducing IVF success rates [2,3]. Preventing or alleviating POA is critical for maintaining oocyte quality. In recent years, as the average age of patients visiting infertility clinics continues to rise, the most urgent issue is improving the IVF success rates using aged oocytes retrieved from advanced-age women. Therefore, research to identify culture media or supplements that can enhance the quality of aged oocytes is of utmost importance. This study demonstrates that EDA significantly improves oocyte quality in both postovulatory-aged and chronologically aged oocytes, enhances embryo developmental capacity in mice, and supports favorable reproductive outcomes in offspring, suggesting its therapeutic efficacy and safety, with potential applications to human IVF programs in the future.

This study evaluated the effects of the antioxidant EDA on oocyte quality using postovulatory-aged oocytes. The use of chronologically aged oocytes is often limited due to the high cost and time required to maintain aged mice (typically older than 12 months), as well as difficulties in obtaining sufficient numbers of aged oocytes. Previous studies have reported that oocytes subjected to POA exhibit multiple age-associated defects, including increased ROS levels, mitochondrial dysfunction, chromosomal abnormalities, reduced fertilization capacity, and adverse effects on offspring development [2,3,18]. These findings suggest that POA serves as a valuable experimental model for studying oocyte aging, providing an alternative to maternal (reproductive) aging. However, not all characteristics of reproductive aging are fully recapitulated in the POA model. Notably, telomere shortening and certain gene expression changes appear to be more specific to chronologically aged oocytes [19,20]. Therefore, the selection between postovulatory and maternal aging models should be carefully considered, depending on the specific phenotype or molecular pathway under investigation.

Meiotic defects, such as spindle abnormalities and improper chromosomal segregation, increase with maternal age, POA, vitrification, or cancer therapy, leading to aneuploidy, infertility, miscarriage, and offspring health issues [4,7,21,22,23,24,25]. Proper spindle assembly and chromosomal alignment are essential for oocyte and embryo quality, and ultimately for ensuring reproductive success and offspring fitness [26]. Antioxidants like auraptene, coenzyme Q10, and melatonin reduce meiotic defects in postovulatory-aged mouse oocytes [4,7,27]. This study shows that EDA supplementation mitigates meiotic defects in both POA and chronologically aged oocytes, with effects comparable to established antioxidants [7,27], indicating its potential to enhance oocyte quality under aging conditions.

Mitochondria are critical for reproductive processes, providing energy for chromosome segregation, spindle assembly, redox balance, and metabolic homeostasis [28]. Furthermore, the fine-tuning of mitochondrial localization and maintenance of adequate ΔΨm in oocytes are closely related to optimal mitochondrial energy production [29]. Previous studies have elucidated that POA disrupts mitochondrial distribution, reduces ΔΨm and ATP levels, and increases ROS, impairing oocyte quality and embryo development [4,6,7,27,30]. In line with previous studies, we found that POA oocytes exhibit reduced mitochondrial activity, decreased mitochondrial ATP production, disrupted spindle organization, and misaligned chromosomes, accompanied by an accumulation of oxidative stress. Importantly, however, EDA treatment increased ATP and ΔΨm, reduced ROS, and supported spindle and chromosome integrity, indicating its role in maintaining redox homeostasis and meiotic apparatus integrity via mitochondrial function.

Oocyte quality directly impacts fertilization and embryonic development, and its decline contributes to impaired female fertility [31]. High-quality oocytes lead to higher fertilization rates, increased blastocyst formation, and enhanced morphological and developmental quality of blastocysts [32]. Unexpectedly, the EDA supplement did not significantly improve fertilization or blastocyst formation rates, though a trend toward improvement was observed compared to the POA group. These findings show discrepancies with prior studies reporting lower rates. Specifically, prior studies reported fertilization and blastocyst formation rates of approximately 40% and 20% [4,5,33,34], while our study observed rates of 62% and 44%, respectively. We speculate that these discrepancies may be attributed to differences in mouse strain, as well as variations in the media used for POA, IVF, and embryo culture. Additionally, several other factors beyond our control—such as oocyte retrieval and handling protocols, the physiological status of females, and sperm quality—may also have contributed to the observed differences.

Embryo development is a complex process governed by the kinetics of the cell cycle and orchestrated by essential molecular and cellular mechanisms. In this context, morphokinetic parameters are important indicators of embryo quality and serve as sensitive measures of developmental potential during in vitro culture [35]. Recently, time-lapse monitoring of embryo development has allowed continuous and non-invasive observation, thereby increasing the accuracy in selecting the embryo with the greatest developmental competence for ART, ultimately leading to improved clinical outcomes [36]. In the present study, EDA supplement was found to improve the quality of postovulatory-aged oocytes; however, its impact on blastocyst formation rates was limited. Given the observed improvement in oocyte quality, we postulated that EDA treatment may similarly exert a beneficial effect on embryo quality, and thus assessed morphokinetic parameters using a time-lapse imaging system. Our research showed that the timing of cleavage, cavitation, and blastocyst formation was delayed in postovulatory-aged oocytes; however, EDA treatment accelerated both morula (tM) and blastocyst formation (tBL). Specifically, the POA group required the longest time to reach the blastocyst stage. Notably, EDA supplementation reduced this developmental delay, resulting in timing comparable to that observed in the young group. Previous studies have reported that tM and tBL parameters of embryos developing into high-quality and euploidy blastocysts are significantly shorter than those in the low-quality and aneuploidy blastocysts [37]. Furthermore, embryos exhibiting delayed developmental progression tend to show diminished developmental potential and increased rates of aneuploidy [38]. Therefore, these results suggest that postovulatory-aged oocytes treated with EDA have a greater potential to develop into high-quality blastocysts.

Antioxidant safety is critical for ART applications [39]. In this study, EDA supplementation did not significantly alter live birth rates or offspring morphology, indicating its safety. Previous studies have reported that POA compromises offspring reproductive fitness, longevity, and behavior [18,40]. Consistent with previously reported defects induced by the POA, female F_1_ offspring from the POA group exhibited fewer MII oocytes, increased meiotic defects, and abnormal mitochondrial distribution, impairing fertility. In contrast, the POA + EDA group showed improved MII oocyte numbers, proper spindle and chromosome alignment, and normal mitochondrial distribution, preserving reproductive potential. Taken together, these results highlight EDA’s safety and efficacy as an antioxidant for ART.

## 5. Conclusions

**Figure 9:**
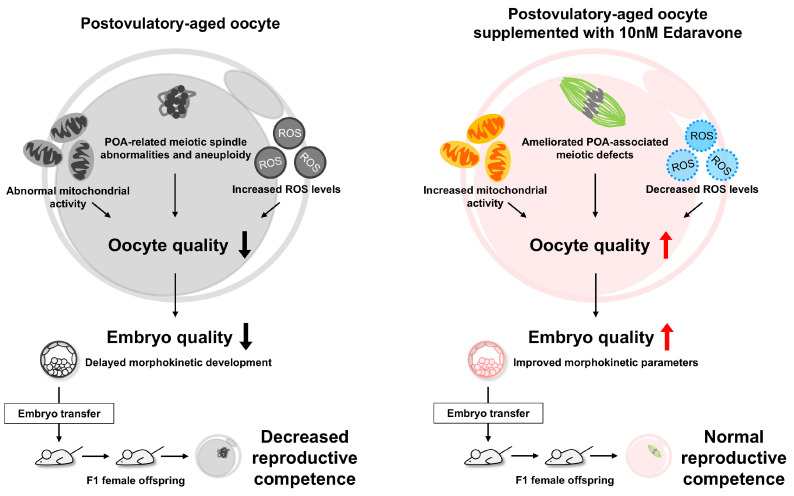
Protective effects of EDA on postovulatory-aged oocytes. We found that EDA supplementation in postovulatory-aged oocytes improves mitochondrial function, reduces ROS levels, and ameliorates POA-associated meiotic defects, thereby enhancing oocyte quality. This improvement promotes better embryo development, as evidenced by enhanced morphokinetic parameters, and leads to normal reproductive competence in F_1_ offspring. Therefore, EDA shows promise as a safe antioxidant strategy to improve oocyte and embryonic competence and ultimately enhance ART outcomes.

This study demonstrates that EDA protects against aging-associated defects in postovulatory-aged oocytes by reducing meiotic abnormalities, enhancing mitochondrial function, and alleviating oxidative stress, thereby improving oocyte quality. Although EDA’s impact on fertilization and blastocyst formation was limited, it improved embryo quality, and offspring exhibited normal reproductive competence (Figure 9). Limitations include the use of mouse models, necessitating further studies on human oocytes and embryos. EDA enhances oocyte and embryonic competence, offering a safe and promising antioxidant strategy to improve ART outcomes.

## Figures and Tables

**Table 1:** Embryonic developmental rates after IVF in the young, POA, and POA + EDA groups.

Group	Total Oocytes	2-Cell (%)	Blastocyst (%) *
Young	302	232 (76.76%)	207 (68.5%) ^a^
POA	213	132 (62.0%)	94 (44%) ^b^
POA + EDA	239	165 (69.0%)	129 (54%) ^ab^

* Different letters indicate significant differences at *p* < 0.05.

## Data Availability

All the data are contained within the article.

## Supplementary Materials

The following supporting information can be downloaded at: https://www.mdpi.com/article/10.3390/antiox14101215/s1, Figure S1: Fertilization and embryonic development of postovulatory-aged oocytes with EDA treatment.

## Abbreviations

The following abbreviations are used in this manuscript:

POA	Postovulatory aging
EDA	Edaravone
ROS	Reactive oxygen species
ART	Assisted reproductive technology
MII	Metaphase II
IVF	In vitro fertilization
ALS	Amyotrophic lateral sclerosis
F_1_	First generation
PMSG	Pregnant mare’s serum gonadotropin
hCG	Human chorionic gonadotropin
COCs	Cumulus–oocyte complexes
GV	Germinal vesicle
IVM	In vitro maturation
IBMX	3-isobutyl-1-methylxanthine
PBS-PVA	Phosphate-buffered saline containing 0.1% polyvinyl alcohol
LAS AF	Leica Application Suite Advanced Fluorescence
ΔΨm	Mitochondrial membrane potential
GSH	Glutathione
dpc	Days post coitum
Mean ± SEM	Mean ± standard error of the mean
