# Supplementary material for: Edaravone Mitigates Postovulatory Aging by Preserving Oocyte and Embryo Quality in Mice
Source: Antioxidants (Basel). 2025 Oct 9;14(10):1215. doi: 10.3390/antiox14101215 (PMC12562146; doi:10.3390/antiox14101215)
Supplement: Supplementary file 1 [file antioxidants-14-01215-s001.zip › antioxidants-3890318-supplementary.pdf]

Edaravone mitigates postovulatory aging by preserving oocyte and embryo quality in mice

Kyeoung-Hwa Kim[1], Eun-Young Kim[1], Ah-Reum Lee[1], Mi-Kyoung Koong[2], Kyung-Ah Lee[1,3,*]

[1] CHA University Global IVF group, Pangyo-ro 335, Bundang-gu, Seongnam-si, Gyeonggi-do, 13488, Republic of Korea

[2] CHA Fertility Center Daegu Station, Dalgubeol-daero 2095, Jung-gu, Daegu, 41936, Republic of Korea

[3] Department of Biomedical Science, Institute of Reproductive Medicine, College of Life Science, CHA University, Seongnam-si, Gyeonggi-do, 13488, Republic of Korea

[*] Correspondence: leeka@cha.ac.kr; Tel.: +82-31-881-7415

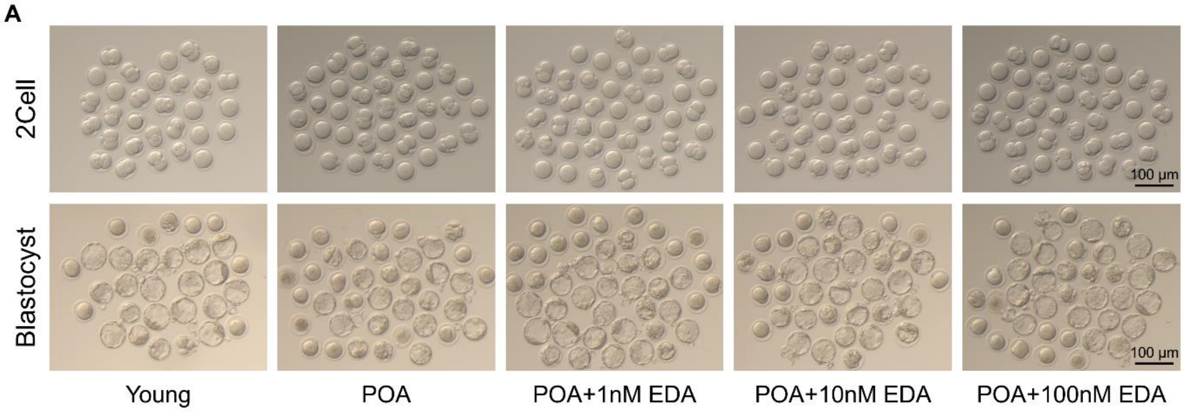

B

Group	Total oocytes	2Cell (%)	Blastocyst (%)
Young	165	117 (70.9)	101 (61.2)
POA	110	55 (50.0)	46 (41.8)
POA+1nM	120	66 (55.0)	54 (45.0)
POA+10nM	126	77 (61.1)	68 (53.9)
POA+100nM	119	66 (55.4)	54 (45.4)

Figure S1. Fertilization and embryonic development of postovulatory-aged oocytes with EDA treatment. (A) Representative images of 2-cell stage embryos and blastocyst embryos derived from the young, POA, and EDA-treated POA oocytes (1 nM, 10 nM, and 100 nM). Scale bars represent 100 μm. (B) Summary table of oocyte counts and percentages of 2-cell and blastocysts stage development across groups. The POA group exhibited reduced developmental competence compared to the Young group, while EDA treatment enhanced 2-cell and blastocyst formation rates in a dose-dependent manner, with optical effects at 10 nM.
